# Balancing Obligation and Autonomy: A Chatbot for Family Caregivers Facing Life-Sustaining Treatment Dilemmas

**DOI:** 10.1093/geroni/igaf122.4360

**Published:** 2025-12-31

**Authors:** Seonghyun Ellin Jeong

**Affiliations:** University of Toronto at St. George Campus, Toronto, Ontario, Canada

## Abstract

Family caregivers often expend excessive energy balancing care, personal life, and emotional stability, especially when illness limits the patient’s autonomy and intensifies ethical dilemmas surrounding the caregiver’s decision making. These dilemmas intensify in the absence of caregiver centered communication and support programs. This project seeks to fill the gap by proposing a self-reflective guiding AI chatbot designed to offer early stage emotional, ethical reflection and informative data before treatment decisions are made. This project builds on an ethical analysis framework that combines principal bioethics to address the moral and emotional costs of choosing life-sustaining treatment. Recognizing the need for early-stage emotional support before a decision is made, we propose a self-reflective AI chatbot prototype to help caregivers articulate their values, emotional reasoning, and potential areas of internal conflict. The chatbot includes a DASS-21-based emotional check-in, CBT-informed narrative prompts, and live-updating local welfare resource information when financial strain is detected. All interactions are anonymous and non-directive. The methodology includes a psychology-based assessment to measure stress levels, experiences of ethical conflict, and financial pressures. Qualitative research will be applied to explore family caregiver’s ethical and emotional pattern, aiming to reveal social and structural foundations in their decision-making process. This study offers a roadmap through an ethical lens for examining real world treatment decisions on behalf of patients. By addressing these systemic challenges and suggesting an intervention model, the research seeks to reduce the risk of unethical decisions among family caregivers, ultimately safeguarding patient rights.

